# COVID-19 relief food distribution: impact and lessons for Uganda

**DOI:** 10.11604/pamj.supp.2020.35.142.24214

**Published:** 2020-08-10

**Authors:** Isabirye Nathan, Musasizi Benon

**Affiliations:** 1 School of Public Health, Makerere University, Kampala, Uganda,; 2 Department of Human Nutrition, Kyambogo University, Kampala, Uganda

**Keywords:** COVID-19, impact, lessons

## Abstract

The COVID-19 pandemic continues to cause uncertainty to Uganda's food security among underprivileged households. The Corona virus Response Team inaugurated a relief food distribution campaign, ensuing from the countrywide COVID-19 lockdown to counter the rising food insecurities in many urban and rural poor households. However, the relief response campaign has received a lot of critics from both rural and urban communities who were planned as the beneficiaries. Three months into the COVID-19 pandemic the population reports; delays in the distribution, poor quality supplies, arrests and continued restrictions, slow paced distribution among household, and a negative impact on the health care system. As a learning from the current experience, we recommend; a multisectoral engagement, better planning, a decentralized food distribution, and formulation of clear food distribution guidelines to guide the future responses. Use of mobile cash transfers to reach out to the food insecure households can support local economies and lower the cost on middlemen and interrelated corruption.

## Commentary

Following the country lockdown presidential directive as a result of COVID-19 pandemic, a relief food distribution campaign was issued on the 25^th^-March -2020 [1]. Uganda's Coronavirus Response Team at the forefront specified on the following measures; that food was to be distributed primarily to the urban poor who are affected by the nationwide lockdown; as a matter of urgency, priority was given to the urban groups in the central region (Capital - Kampala and neighbouring Wakiso district); the beneficiaries being small business operators who earn hand to mouth, the elderly, sickly, and lactating mothers; rations to include 6kg of maize flour per person, 3kg of beans per person, and salt (it was slated that lactating mothers and the sick were to receive 2kgs of powdered milk and 2kgs of sugar). Other districts of Uganda were scheduled to be reached depending on the resources mobilised during the response campaign. As a measure to cab the spread of the virus, security forces were given the responsibility to undertake the household-based food distribution exercise. It was designated to be house to house, door to door, as a measure to avoid crowding and to maintain social distancing [1].

Many Ugandans have criticized the design of the food relief strategy with predicted ambiguities [2,3]. At more than three months into the COVID-19 pandemic, the government food support strategy seems to have a slow bearing. Primarily, the food relief program projection of 1.5 million as the number of people who will need food support was an underestimation. Majority of vulnerable households have not benefited from the food donation exercise, including many urban poor and rural dwellers scattered in different regions of Uganda. The unpredicted end to the lockdown, continued restrictions and muddle continue to suppress the relief food program and the livelihood of the low socio-economic class. The government has continued to mobilize relief donations from stakeholders and urged individuals to make food donations to those at risk within the neighbourhood. Also, the supply of high-quality food staffs especially posho and beans is being compromised as a result of aflatoxin, soil, sandy particles and other physical impurities in food because of poor crop methods in the gardens and post-harvest handling at drying, sorting, storage, processing and packaging [4]. The lack of clear guidelines on quality and the process of contribution seems to play against the pooling of resources and distribution of donations. The result has been unceasing arrests, restrictions and threats to charge whoever is found distributing relief food in the community. Similarly, stories reported on the local media have revealed that quality of care for people living with HIV and expectant mothers has been negatively impacted by the lockdown- in terms of adherence to treatment. Poor access to food still plaques majority of Ugandans living especially in the rural and unevenly distributed by region [5]. Uganda is ranked among the low-income food-deficit countries, and the most affected region being Karamoja, Bukedi and Busoga region. Putting into consideration the contributors to food insecurity (lack of choice, anxiety of running out of food, forced changes in preferred eating habits) due to economic constraints [6] and the disruption in routine as a result of the COVID-19 pandemic, the population at risk turns out to be scattered countrywide.

To better tackle the food relief response exercise, below is what we recommend; (i) Office of the prime minister alone is not enough-in such nationwide food relief response. Now that Uganda has a well-established decentralised system right from the district level to the village setting. It is therefore important to consider using the already established systems to undertake nationwide food distribution campaigns. This can be further strengthened by the use of an already grounded system such as local council representatives and Village Health Teams (VHTs) during the food distribution planning process. Currently, the local council office bears an updated record of the welfare status of its community members at a village level, and the VHTs have the responsibility of mobilization of village members for community health activities. In that way, they are conversant of the settings where they operate and belong especially in slums and rural communities. Consultation of the above systems henceforth during the design and implementation of relief food programs during similar epidemics is very key. This will ease identification and making right estimations of households that are at a higher risk of becoming food insecure and the engagement of stakeholders. It will also allow the security forces to remain with their role of provision of security during such emergencies [7] (ii) involvement of the line ministries (Finance, agriculture, health, research institutions) through a multisectoral approach will enable effective planning of food distribution, price restrictions of food items and inspection at a national level (iii) proper planning ought to utilise the COVID-19 disease spread model to estimate the speed and pattern, both geographically and overtime to design appropriate food distribution plans (iv) It is important to plan as a country and rejuvenate the regional stock centers for food in silos countrywide to avoid depending on donations during a nationwide emergency (v) During a food relief response to a countrywide emergency such as this, clear standard operating procedures and measures should be communicated to guide the entire process. One of the avenues would be the use of mobile cash transfers to the food insecure urban and local residents during pandemics such as COVID-19. This is a tool that can be fast, support local economies but also reduces the cost on middlemen and related corruption.

## Conclusion

Uganda's food insecurity remains classified as serious. Food relief consideration in a global crisis such as the COVID-19 pandemic ought to be planned well to cater for all pockets of stress due to hunger among underprivileged households scattered countrywide.
